# Genetic and epigenetic engines of diversity in pathogenic microbes

**DOI:** 10.1371/journal.ppat.1006468

**Published:** 2017-09-14

**Authors:** R. Blake Billmyre, Joseph Heitman

**Affiliations:** Department of Molecular Genetics and Microbiology, Duke University Medical Center, Durham, North Carolina, United States of America; McGill University, CANADA

## Introduction

**Fig 1:**
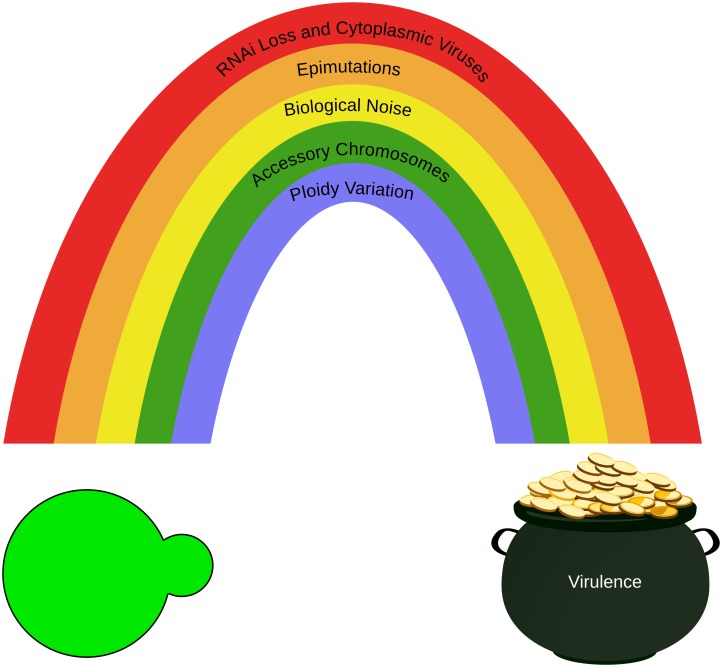
Multiple pathways to phenotypic diversity lead to virulence. Evolution can take any of multiple routes to enhance fitness and, in the case of pathogens, virulence. Here, we depict the 5 mechanisms of adaptation that we discuss in this review, which are only some of the myriad pathways available to microbes.

Mutation is traditionally thought of as the raw material for evolution, but it is not the only medium upon which selection acts. Microbial pathogens deploy a panoply of strategies to adapt and thrive when exposed to diverse environmental stresses. Previously, we reviewed some of these strategies [1], and here, we update and expand on these to include RNA interference (RNAi) loss and cytoplasmic elements, epimutations, biological noise, dispensable B chromosomes, and variation in ploidy states (Fig 1). Microorganisms are capable of traversing multiple routes to reach an evolutionary peak, even those that are often deleterious from the perspective of either an unstressed microbe or a multicellular organism.

### RNAi loss in model and pathogenic microbes

Viruses are typically thought of as deleterious and many eukaryotic microbes contain an active RNAi system to defend themselves from viral assault. However, RNAi pathways have been repeatedly and independently lost across the eukaryotic kingdom, including in the model yeast *Saccharomyces cerevisiae* [2], the human fungal pathogen *Cryptococcus deuterogattii* [3], the plant fungal pathogen *Ustilago maydis* [4], and the Old World *Leishmania* parasites of humans and other animals [5]. Some RNAi losses are correlated with double-stranded RNA (dsRNA) viral infection, and within fungi, these viruses appear to be beneficial, as many produce toxins that kill neighboring related and unrelated yeasts [6]. This inherently provides an advantage to the microbe in competitive situations, such as in accessing nutrients. In the clinical setting, toxin-encoding viruses could have profound effects by modulating the microbiome of an infected site, allowing the pathogen to establish infection. In *Leishmania* parasites, paradoxically, viruses are present in the RNAi-proficient lineage, in which they trigger induction of host toll-like receptor 3 (TLR3) (dsRNA) and toll-like receptor 7 (TLR7) (single-stranded RNA [ssRNA]) responses during infection, resulting in more severe mucocutaneous infections instead of the typical cutaneous infections [7].

Cytoplasmic viral elements can contribute to phenotypic variation in less obvious ways, as well. The presence of dsRNA viruses modulates the phenotype of numerous mutations in yeast, including both gene deletions and strain-specific mitochondrial polymorphisms [8]. For example, *SKI7* is essential in the presence of dsRNA viruses but otherwise dispensable. Nuclear-cytoplasmic interactions may explain some of the “missing” heritability observed for many quantitative traits.

### Epimutation allows transient silencing of temporarily disadvantageous genes

One type of stress encountered by microbes involves chemical signals that act on or through microbe-encoded proteins, such as antimicrobials that rely upon hijacking microbial machinery for maturation, toxicity, or uptake. Microbes can escape this type of chemical stress by inactivating a drug target gene if it is not essential for microbial viability. Mutations readily inactivate genes but are difficult to revert to restore wild-type function if the stress is transient. One alternative route involves epimutation, or a heritable change that does not alter DNA sequence, to transiently inactivate the gene. In *Mucor circinelloides*, the RNAi pathway can silence the *fkbA* gene, which encodes FKBP12, the target of both FK506 and rapamycin, in a reversible fashion [9]. RNAi-dependent epimutation is repressed by 2 of the 3 endogenous RNA-dependent RNA polymerase (RdRP) genes, which suggests the frequency of epimutation could be regulated by environmental cues that impact expression or function of individual RdRP genes [10]. These 2 RdRP genes are responsible for a noncanonical dicer-independent mRNA degradation pathway [11], which may compete with epimutation. Similarly, in the plant pathogen *Phytophthora sojae*, small RNA (sRNA)-dependent gene silencing can inactivate the *Avr3a* effector. This effector is recognized by host plants with the *Rps3a* R gene, resulting in host immunity. Consequently, pathogens that silence *Avr3a* are enabled to successfully infect otherwise resistant hosts, expanding their host range [12]. A recent follow-up study suggested that heritable silencing may be specific to particular genetic backgrounds, as crosses between silenced strains and unsilenced siblings produced silenced progeny, but outcrosses between a silenced strain and a more distantly related partner produced predominantly unsilenced progeny, even when the *Avr3a* sequence was identical [13].

Epimutation also generates phenotypic variation in the human parasitic pathogen *Plasmodium falciparum*, the etiological agent of malaria. *P*. *falciparum* is adapted to grow in multiple disparate host environments throughout its life cycle, including the salivary glands of mosquitos, human hepatocytes, and human erythrocytes. *Plasmodium* parasites are able to generate diversity by randomly and epigenetically silencing portions of redundant gene sets in a process called clonally variant gene expression (CVGE) [14]. As a result, within a genetically clonal population, multiple invasion approaches are employed, making it much more difficult for a host to adapt to prevent invasion through any one pathway. CVGE is mediated by histone 3 lysine 9 trimethylation (H3K9me3) of silenced genes, resulting in their localization to the periphery of the nucleus [15]. Epigenetic changes also mediate resistance to chemical stresses like blasticidin in *Plasmodium*, with loss of the activating marks histone 3 lysine 9 acetylation (H3K9ac) and histone 4 lysine 4 trimethylation (H3K4me3) from the promoter regions driving silencing of the 5-member *clag* gene family and eliminating blasticidin toxicity [16]. Regulatory epimutations may underlie many transient phenotypes that enable pathogens to vary their phenotypic repertoire to cause disease.

### Biological noise generates diverse phenotypic states from genetically identical populations

Biological noise introduces variation into a population of cells that is independent of any intrinsic genetic differences. While most biological systems are robustly buffered, small differences in expression level are possible between even genetically identical individuals in identical environments. This variability is referred to as intrinsic noise and is the result of stochastic events during transcription of a gene as opposed to extrinsic noise within a locus, which results from noise in upstream signaling pathways or other population-level heterogeneity [17]. Noise is particularly impactful for proteins present in low quantities in cells, which may vary, for example, between zero or one copy per cell, such as in Ada-dependent DNA alkylation damage sensing in *E*. *coli*, in which cells produce an average of 1 copy of the protein per cell division, with 20% to 30% of cells containing no Ada protein at any given time [18]. This results in dramatic differences between siblings in a population, with some individuals able to rapidly sense and respond to alkylation damage and others reliant upon eventual translation of Ada to do so.

Population-level diversity can be important during infection. In *Salmonella typhimurium*, induction of interferon responses is dependent upon the level of PhoPQ expression in the invading bacteria, which is variable between individual bacterium [19]. Noise can also mediate antibiotic survival through regulation of persistence pathways like the toxin-antitoxin pathway. Stochastic increases in toxin expression alter the stoichiometric relationship between the toxin and the antitoxin, triggering transient growth arrest and consequent antibiotic resistance in *E*. *coli* [20]. Similarly, variation in quantity of the catalase-peroxidase KatG drives persistence to the drug isoniazid in *Mycobacterium smegmatis* [21]. Because KatG converts isoniazid into a toxic compound, cells that transiently lack KatG can survive antibiotic exposure and survive to replicate. Periodic KatG expression results in the death of a portion of cells, but enough cells lack KatG at any given time to generate a steady-state population that can persist until drug challenge is removed or potentially until resistance evolves.

Notably, pathogens employ noise to adopt self-sacrificing altruistic behaviors that would otherwise be negatively selected if genetically encoded. For example, during *S*. *typhimurium* infections, a subset of the bacteria stochastically express a type III secretion pathway and invade the gut epithelium, triggering inflammation that clears the intestinal microbiota. Most of the invasive bacteria are killed by host defenses, but the host response enhances survival of the genetically identical noninvaders [22]. Because this behavior is the result of noise, the surviving noninvasive bacteria can give rise to new invading populations during subsequent infections. This type of kin selection may underlie many altruistic behaviors during infections, such as in the fungal pathogen *C*. *deuterogattii*, in which some individuals engulfed by macrophages undergo slowed growth and form tubular mitochondria, which protects their siblings with nontubular mitochondria from killing when they are present in the same macrophage [23].

### “B” accessory chromosomes allow shifts in host range

Organisms utilize a core set of genes contained within a set of nondispensable chromosomes found in all individuals of a species. However, in addition to this set of “A” chromosomes, some organisms harbor 1 or more “B” chromosomes. These dispensable chromosomes typically lack genes with essential functions and are often deleterious and selfishly inherited. As a result, individuals within a species harbor varying sets of B chromosomes, with variation in both the presence and copy number of these selfish elements. Often, these chromosomes are dominated by noncoding sequence and repetitive elements. In some ways, the selfish nature of B chromosomes is akin to that of bacterial plasmids that utilize toxin-antitoxin systems to ensure their maintenance. In most multicellular eukaryotes, these B chromosomes are disadvantageous.

However, in fungi, the presence of B chromosomes, variously referred to as supernumerary, dispensable, accessory, or lineage-specific chromosomes [24], can be advantageous. Fungal B chromosomes are more gene rich than plant and animal B chromosomes and can play key roles in virulence and adaptation. For example, B chromosomes in *Fusarium oxysporum* confer pathogenicity and allow expansion of host range [25]. These chromosomes can transfer between closely related *Fusarium* strains, and as a result, virulence to a particular plant species is a polyphyletic trait. Dispensable chromosomes are found in many plant pathogenic fungi, including the wheat pathogen *Zymoseptoria tritici*, which contains as many as 8 dispensable B chromosomes [26], and *Nectria haematococca*, in which a specific B chromosome enables growth on pea plants [27–29].

One hypothesis to explain the high frequency of supernumerary chromosomes in plant pathogens and their dearth in animal pathogens is that it may represent adaptation to the effector-specific immunity found in plants. B chromosome–encoded effector suites found in organisms like *F*. *oxysporum* can be transferred to new individuals to expand their host range [25]. However, when these pathogens move to a host in which one of these effectors is recognized by a host R gene triggering immunity, the Avr gene could be readily lost in a portion of the pathogen population via error-prone segregation of the B chromosome. This could then enable a restoration of virulence and a loss of pathogen recognition by the host.

### Ploidy variation enables global changes in phenotype

In eukaryotic pathogens, variation in ploidy also generates phenotypic diversity. Many fungi are capable of growing in either haploid (1N) or diploid (2N) states. Experiments in *S*. *cerevisiae* have shown that an even higher increase in ploidy to tetraploidy (4N) accelerates the rate of adaptation by buffering altered alleles and even unleashing mutations that are specifically beneficial in the tetraploid state [30]. Furthermore, increases in ploidy moderate the effects of losing or gaining a single chromosome, allowing higher rates of aneuploidy, which can itself provide further phenotypic variation and allow further mutation. Variation in ploidy even generates de novo phenotypic variation in isogenic isolates. Comparison of *S*. *cerevisiae* and *S*. *paradoxus* reveals that diploids outcompete haploids under some growth conditions yet are inferior under other conditions, and that these ploidy advantages are conserved between *S*. *cerevisiae* and *S*. *paradoxus* [31]. This suggests that selection acts either on exposure to specific stresses during different portions of the life cycle or that the preferred ploidy of different yeast strains may differ based on the environmental stresses they encounter. Perhaps the most extreme example of ploidy variation occurs in the human fungal pathogen *C*. *neoformans*, in which enlarged, polyploid titan cells develop in the lungs during infection that are up to 20x larger in diameter and up to 312C in genome content [32,33]. Titan cells produce haploid daughters, but under stress conditions such as treatment with the antifungal drug fluconazole, they can produce aneuploid daughters that display phenotypic variability [34].

## Conclusions

Here, we have highlighted some of the myriad mechanisms by which microorganisms as diverse as fungi, oomycetes, bacteria, and parasites generate phenotypic diversity to better exploit their environments and survive extreme stresses. Both pathogens and their hosts take part in a perpetual evolutionary arms race. Each adapts to tip the balance in their own favor, but each also faces unique biological constraints. As a result, eukaryotic hosts evolve layers of complexity to their defenses, while pathogens discard the rule book and alter their phenotype by any and all means available.
